# Prognostic value of CD44v9 expression in human cancers: A systematic review and meta-analysis: Retraction

**DOI:** 10.1097/MD.0000000000024450

**Published:** 2021-01-22

The article, “Prognostic value of CD44v9 expression in human cancers: A systematic review and meta-analysis”,^[1]^ which appears in Volume 99, Issue 30 of *Medicine*, is being retracted after authors reported issues with data and misleading definitions. The authors failed to include two studies, both which showed the prevalence of positive CD44v9 expression. One of the studies not included also presented association between CD44v9 expression and CSS (cancer-specific survival), which influenced the pooled prevalence of positive CD44v9 expression and the association between CD44v9 expression and CSS. Additionally, authors defined OS (overall survival), DSS (disease-specific survival), and CSS (cancer-specific survival) as the same, affecting the overall results of the study.
